# Sensorimotor Synchronization in Healthy Aging and Neurocognitive Disorders

**DOI:** 10.3389/fpsyg.2022.838511

**Published:** 2022-03-17

**Authors:** Andres von Schnehen, Lise Hobeika, Dominique Huvent-Grelle, Séverine Samson

**Affiliations:** ^1^Université de Lille, ULR 4072 – PSITEC – Psychologie: Interactions, Temps, Emotions, Cognition, Lille, France; ^2^Sorbonne Université, Institut du Cerveau - Paris Brain Institute - ICM, Inserm, CNRS, APHP, Hôpital de la Pitié Salpêtrière, Paris, France; ^3^Hôpital Gériatrique les Bateliers, Pôle de Gérontologie, CHU Lille, Lille, France; ^4^Epilepsy Unit, AP-HP, GHU Pitié-Salpêtrière-Charles Foix, Paris, France

**Keywords:** aging, dementia, rhythm, finger tapping, timing, Alzheimer’s disease, music, neurodegenerative diseases

## Abstract

Sensorimotor synchronization (SMS), the coordination of physical actions in time with a rhythmic sequence, is a skill that is necessary not only for keeping the beat when making music, but in a wide variety of interpersonal contexts. Being able to attend to temporal regularities in the environment is a prerequisite for event prediction, which lies at the heart of many cognitive and social operations. It is therefore of value to assess and potentially stimulate SMS abilities, particularly in aging and neurocognitive disorders (NCDs), to understand intra-individual communication in the later stages of life, and to devise effective music-based interventions. While a bulk of research exists about SMS and movement-based interventions in Parkinson’s disease, a lot less is known about other types of neurodegenerative disorders, such as Alzheimer’s disease, vascular dementia, or frontotemporal dementia. In this review, we outline the brain and cognitive mechanisms involved in SMS with auditory stimuli, and how they might be subject to change in healthy and pathological aging. Globally, SMS with isochronous sounds is a relatively well-preserved skill in old adulthood and in patients with NCDs. At the same time, natural tapping speed decreases with age. Furthermore, especially when synchronizing to sequences at slow tempi, regularity and precision might be lower in older adults, and even more so in people with NCDs, presumably due to the fact that this process relies on attention and working memory resources that depend on the prefrontal cortex and parietal areas. Finally, we point out that the effect of the severity and etiology of NCDs on sensorimotor abilities is still unclear: More research is needed with moderate and severe NCD, comparing different etiologies, and using complex auditory signals, such as music.

## Introduction

Sensorimotor synchronization (SMS) is defined as temporal coordination of a motor rhythm with an external rhythm. It is a form of adaptive interaction with the environment (Schwartze et al., 2011). Being able to synchronize to regularities in temporal structure and matching one’s movements to those of others is of obvious importance in activities whose essence is based on creating a shared temporal structure, such as dance or joint music making (Sebanz et al., 2006). However, organizing one’s own behavior according to the dynamic unfolding of events in the environment is crucial to many more situations than that. Interpersonal entrainment is a key rhythmic feature in human interactions, including non-musical interactions (Bispham, 2006). Many situations that do not require synchrony nonetheless cause people to synchronize their movements to each other, such as when people unintentionally synchronize their postural sway (Shockley et al., 2003) or lower limb movements while walking (van Ulzen et al., 2008; Nessler and Gilliland, 2010), or entrain the frequency of their movements to each other while clapping hands. While the use of a pulse in structuring one’s behavior in time is self-evident in musical activities in which the goal is to maintain temporal stability, a pulse also appears in a more loose and subconscious way in interpersonal turn-taking interactions (Bispham, 2006). Spoken language contains remarkable temporal regularities in the signal envelope of the produced acoustic signal, as well as in vocal tract movements and syllable duration and rate (Poeppel and Assaneo, 2020). When comparing linguistic groups, correlations between aspects of temporal structuring in music emerge (Patel and Daniele, 2003) underscoring the universality of attending to regularities in auditory signals generally. Dynamic attending theory (Jones, 1976; Large and Jones, 1999) proposes that when presented with an auditory sequence, listeners’ attention will oscillate periodically such that it is higher on the beat than off the beat, to allow for optimal processing and forming the basis for prediction. Although dynamic attending theory has mainly been used to explain phenomena of rhythmic perception in relation to sequences that are musical or isochronous (i.e., periodical with a constant interval between beats), it has also been proposed to explain interactional synchrony between people that happens in a less strictly rhythmic fashion (Cason et al., 2017). While language does not follow an isochronous rhythm, it is nonetheless based on temporal regularities, facilitating understanding by allowing the listener to predict incoming auditory input (Byrd and Saltzman, 2003; Port, 2003). In a domain-general fashion, entrainment to regular auditory input may thus enhance the representation of regularities in sound, guiding one’s attention to point in time at which meaningful information is being delivered (Obleser and Kayser, 2019; Mathias et al., 2020). Accordingly, the perceptual system appears to be tuned to the natural rhythm of speech (frequencies between 2 and 8 Hz; Poeppel and Assaneo, 2020) Contrariwise, problems to perceive regularities in the environment and tune one’s attention to them might hamper interpersonal communication. Indeed, it has been demonstrated that an inability to perceive a beat in music generalizes to a weakness in perceiving periodicities in speech (Lagrois et al., 2019). In other words, measuring a person’s ability to perceive and synchronize with rhythms in the environment does not only reveal that person’s sense of rhythm, but a more general propensity to predict events in the environment and relate to others.

It is therefore worthwhile to assess and study SMS abilities. However, the effect of age and neurocognitive disorders (NCDs) on sensorimotor abilities remains understudied. NCDs are a growing health concern to which an effective treatment remains elusive. Besides cognitive decline and degradation of memory performance, a common consequence of this condition is a decrease in the quality of social relationships. Decreased interactions with others and the environment is common to patients with dementia (Colling, 2000), a potential result of sensory decline common to old age (Gates and Mills, 2005; Correia et al., 2016) and to NCDs (Armstrong, 2009; Hardy et al., 2016; Brenowitz et al., 2019), or a consequence of living in isolation or in an inpatient context with insufficient social stimulation (Chung, 2004; Kolanowski et al., 2006). Nonetheless, this impairment in communication might also be understood as an impairment in the detection of regularities in the environment (Hoehl et al., 2021). Stimulating and training someone’s sensorimotor abilities might inadvertently restore their capacity to predict events in the environment, and to relate successfully to others.

Music-based interventions, which are increasingly suggested in the treatment of NCDs [Guideline Adaptation Committee, 2016; National Institute for Health and Care Excellence (NICE), 2019], might reach their peak of effectiveness if they successfully stimulate SMS (Ghilain et al., 2019; Hobeika and Samson, 2020), particularly in those individuals whose motor abilities remain relatively unimpaired despite decline in other domains. Music-based interventions exist in many forms but those that encourage active participation appear to bring greater benefits on behavioral and psychological variables than those in which patients listen passively (Sakamoto et al., 2013; Särkämö et al., 2014), suggesting an important role of SMS in the effectiveness of these interventions. First, moving in time with others has been shown to promote feelings of social cohesion, prosocial attitudes, and cooperative behavior (Wiltermuth and Heath, 2009). Second, temporal expectations elicited by the perception of a musical beat may stimulate the reward network and induce pleasure (Salimpoor et al., 2011). Improving a person’s temporal prediction abilities might help them synchronize and interact with others (Pecenka and Keller, 2011), and therefore improve communication and reduce isolation.

At present, motor abilities have been well examined in patients with Parkinson’s disease (Grahn and Brett, 2009; Dalla Bella, 2018) but studies investigating SMS skills in other neurological diseases (notably NCDs) are scarce. Even in the case of healthy physiological aging, little consensus exists in the literature as to whether sensorimotor skills are preserved in old age. With this review, we aim to shed light on the question of how SMS abilities develop in the late decades of life and over the course of NCDs. Additionally, we touch upon the possibility of using SMS as a diagnostic tool. Since NCDs are afflictions of the aging brain, it is important to disentangle NCD-related changes in SMS from those related to healthy aging. Throughout this review, we will use the terms major and mild NCD. Major and mild NCD are the current terms used in the *Diagnostic and Statistical Manual of Mental Disorders* (5th ed.; DSM-5; American Psychiatric Association, 2013) for what is otherwise referred to as dementia and mild cognitive impairment (MCI). Although technically NCD is a somewhat broader term, for example including cognitive impairment in young people caused by traumatic brain injury or HIV infection, we will use the term NCD when discussing studies that themselves might have used the terms dementia or MCI in their nomenclature. In any case, all studies about NCDs reviewed here involved elderly subjects. Finally, Parkinson’s disease is often discussed in the context of NCDs, and indeed “major/mild NCD [possibly] due to Parkinson’s disease” exist as conditions in the DSM-5. However, Parkinson’s disease is primarily a movement disorder, and as such, there already exists a bulk of literature regarding motor abilities in this population (Grahn and Brett, 2009; Grabli et al., 2012) as well as the use of rhythmic stimulation in rehabilitation (Dalla Bella et al., 2017a; Cochen De Cock et al., 2018; Dalla Bella, 2018), so we do not wish to add to this literature and restrict our review to the other, mostly cortical, etiologies of NCD, such as Alzheimer’s disease (AD), vascular NCD, or NCD with multiple etiologies.

Our review is therefore organized as follows: First, after clarifying what is meant by SMS and how it is quantified and assessed, we present the cognitive and brain mechanisms that lie at the heart of SMS, in general terms and as a function of the more specific task requirements. Next, we give an overview of how the aging process influences cognitive performance and brain structure and function, followed by a section on how these processes are influenced by NCDs. In both cases, we offer some predictions with regard to SMS performance. We then review the available literature on SMS in healthy aging, followed by the literature on SMS in NCDs to evaluate the predictions we made. We conclude with some suggestions for future research.

### Sensorimotor Synchronization: Common Paradigms and Ways of Assessing Performance

Synchronizing one’s movement with an external rhythm can come in many forms including moving one’s limbs with an auditory sequence, walking, or dancing (Repp and Su, 2013). However, the most commonly employed paradigm is that in which a participant is asked to produce regular, rhythmic taps with a finger. In the context of this review, we refer to two main types of tapping paradigms as unpaced tapping and paced tapping. Unpaced tapping corresponds to tasks in which participants produce movements at a self-generated rate, very often to identify someone’s spontaneous motor tempo (SMT) but also to assess how fast or slow one can maintain a regular rate. Paced tapping, on the other hand, consists of synchronizing one’s tapping to an auditory pacer. This pacer can be a simple isochronous (metronomic) sequence, with the goal to match each tap to the onset of each beat, but it can also be a more complex stimulus (such as music) where the beat must be inferred. Very often, the participant continues to do this until the signal fades, a case to which we refer as *synchronization without continuation*, to distinguish it from *synchronization–continuation*, that is, tasks in which participants first synchronize their tapping with an auditory pacer, but then continue tapping at the same speed for some time after the stimulus has faded.

Sensorimotor synchronization performance measured by these tasks is described in terms of variability and accuracy. When the participant synchronizes to an external metronome or rhythmic stimulus, accuracy or beat alignment refers to the extent to which taps occur before (negative asynchrony) or after (positive asynchrony) the beat (event onset in the external rhythm) and is thus expressed as a difference in ms. Variability or precision refers to the standard deviation of the asynchronies. On the other hand, during continuation tapping, the main observable is the inter-tap interval (ITI), and its average and standard deviation are reported. Mean ITI reflects whether the subject drifts away from the original tempo, while ITI variability, or precision, refers to how consistently an individual’s taps are distributed around a period and is given by the standard deviation (SD) of the ITIs within a trial, or the coefficient of variation (CV; SD divided by mean ITI). Sometimes, variability is expressed as its inverse, consistency. Finally, mean ITI (reflecting average speed) and ITI variability are also used as outcomes in self-paced tapping, in the absence of an external stimulus.

## Brain and Cognitive Mechanisms Involved in Sensorimotor Synchronization

**Figure 1:**
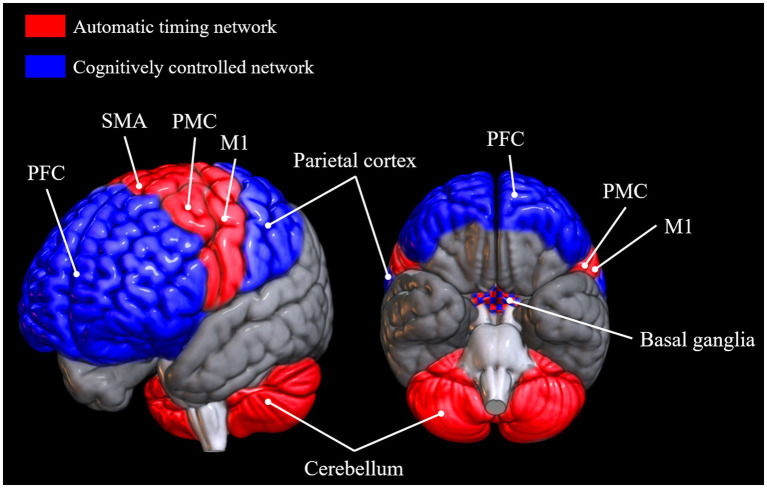
Brain areas reported to be active in tasks requiring automatic timing and cognitively controlled timing, respectively (Lewis and Miall, 2003; Buhusi and Meck, 2005; Koch et al., 2009; Coull et al., 2011, 2013; Repp and Su, 2013). Brain networks were plotted onto a standard MNI152 template rendered with the open-source software MRIcroGL (McCausland Center for Brain Imaging, University of South Carolina). PFC, prefrontal cortex; SMA, supplementary motor area; PMC, premotor cortex; M1, primary motor cortex.

Which brain networks are involved in SMS tapping depends on the exact nature and instructions of the task, but very generally, areas involved in tapping tasks are primary sensory and motor cortices, supplementary motor area (SMA), anterior cerebellum, dorsolateral prefrontal cortex, premotor cortex, parietal areas, and the basal ganglia (Larsson et al., 1996; Rao, 1997; Penhune and Doyon, 2005; Molinari et al., 2007; Chen et al., 2008; Witt et al., 2008; Kung et al., 2013; Repp and Su, 2013). Another general observation that can be made across a variety of timing tasks is that there seem to be two somewhat disparate networks for processing intervals depending on the length of the inter-onset interval (IOI; Repp and Su, 2013). The automatic timing network is involved in the processing of sub-second intervals and includes the primary motor cortex, the SMA, the premotor cortex, and the cerebellum (Lewis and Miall, 2003). The cognitively controlled timing network is involved in the processing of intervals longer than 1 s. It comprises areas of the parietal cortex, prefrontal cortex, and the basal ganglia (Lewis and Miall, 2003; Buhusi and Meck, 2005; Koch et al., 2009; Coull et al., 2011, 2013; Figure 1).^1^ The basal ganglia, universally involved in beat processing (Grahn, 2009), are also considered part of the automatic timing network by some (Lewis and Miall, 2003; Koch et al., 2009), but not all authors (Buhusi and Meck, 2005), although, as Coull et al. (2011) point out, it may well be that different nuclei within the basal ganglia are responsible for timing in the sub- and supra-second range, respectively. Contrary to the automatic timing network, the cognitively controlled timing network is based on brain areas involved in high-level cognitive functions. Indeed, Coull et al. (2011, 2013) suggest that explicit estimation of current stimulus duration is a conscious cognitive operation necessary to perceive periodicity in a slow stimulus with an interval length of above around 1 s. In a related manner, for the successful perception of and synchronization to a slow beat, people might employ tactics, such as explicit counting (Grondin et al., 1999) and/or subdivision of the given interval (Repp, 2003; Repp and Doggett, 2007), strategies that arguably rely more on attentional and memory processes and corresponding brain networks and less on pure more mechanisms and structures. According to Koch et al. (2009), the dorsolateral prefrontal cortex’s role in the timing of long intervals might be related to WM. A study by Henley et al. (2014) supports this idea, as they observed correlations between the ability to maintain a slow beat and their WM capacity, measured with the digit span backwards test (Wechsler, 1981).

### Unpaced Tapping

As mentioned in the previous section, unpaced tapping tasks are frequently in such a manner that one’s natural, SMT is measured. SMT tends to be consistent across repeated measures within the same participant and is seen as a reliable metric of internal tempo or clock (Denner et al., 1964). In addition, one might look at preferred perceptual tempo, which is a perceptual analogue to SMT.

In cognitive terms, results of spontaneous tapping studies have been explained in terms of an internal clock mechanism. Many authors have argued that people possess an internal clock which determines not only at which tempo they comfortably synchronize their movements, but also their ability to predict how events in the environment unfold over time (McAuley et al., 2006; Turgeon and Wing, 2012). Such an internal clock is often conceptualized as a pacemaker emitting pulses and a reference memory evaluating time by counting the number of pulses emitted (Church, 1984).

Some evidence suggests that tapping at a self-determined speed, as opposed to synchronizing one’s movement with an external pacer, engages particularly the primary motor cortex, premotor cortex, SMA, dorsolateral prefrontal cortex, as well as areas of the parietal lobe and cerebellum (Larsson et al., 1996; Witt et al., 2008). Furthermore, a lesion study involving patients with basal ganglia pathology also demonstrated that self-paced tapping may depend on basal ganglia integrity, as patients tapped at more heterogeneous rates and with increased variability compared to healthy controls (Schwartze et al., 2011). The authors posit that self-paced tapping might specifically depend on the integrity of connections between pre-SMA and striatum.

Besides paradigms to identify a person’s SMT, unpaced tapping can also take the form of tasks in which participants tap as fast or as slow as possible while maintaining a continuous regular rhythm. Tapping at a fast rate might engage the same automatic timing network, but might additionally depend on factors like the time required for muscle contractions and muscular and joint flexibility (Daley and Spinks, 2000; Carmeli et al., 2003). However, synchronization precision at a very fast tempo might additionally require attentional resources, as suggested by a study that found a correlation (Colella et al., 2021) between variability of tapping and score on the Frontal Assessment Battery, a test of frontal lobe integrity said to reflect attention (Dubois et al., 2000). Tapping at a very slow rate, on the other hand, might involve a conscious representation of the current interval and a memory aspect, likely implicating structures from the cognitively controlled network (Figure 1).

### Paced Tapping

As mentioned above, we distinguish between simple synchronization tasks without continuation and synchronization–continuation paradigms. The former type of task is expected to engage the motor circuitry associated with timing tasks, such as motor cortical areas, cerebellum, and basal ganglia, with some studies suggesting even stronger involvement of the premotor cortex and cerebellum with such externally paced movement than when a tempo is internally maintained (Del Olmo et al., 2007; Kornysheva and Schubotz, 2011), like in the continuation phase of synchronization–continuation tasks. While a few studies suggest the same areas to be involved in continuation as in synchronization (Jäncke et al., 2000; Jantzen et al., 2004), continuation tapping after an external stimulus has faded might elicit additional activation in primary sensory and motor cortices (Gerloff et al., 1998), premotor cortex, SMA (Serrien, 2008), thalamus, and basal ganglia (specifically, putamen; Lewis et al., 2004). One study also demonstrated that a prefrontal–parietal–temporal network, containing the dorsal and ventral prefrontal cortex, middle temporal gyrus, and parietal lobes, may be especially activated during continuation tapping (Jantzen et al., 2007). The authors suggest that the involvement of the prefrontal cortex reflects the task’s requirement to form an internal representation of the sequence tempo and to recruit attentive processes. This is supported by a study finding prefrontal white matter integrity to be related to variability of tapping in the continuation phase of a synchronization–continuation task (Ullén et al., 2008).

Just like for unpaced tapping, the exact involvement of brain networks and cognitive mechanisms may vary depending on sequence tempo in tasks involving synchronization with and without continuation alike. Accordingly, we can expect particular recruitment of structures like the prefrontal cortex and the parietal lobes (the cognitively controlled timing network) in paced tapping at speeds beyond 1 s (Koch et al., 2009).

## Brain and Cognitive Correlates of Healthy Aging and Neurocognitive Disorders

### Healthy Aging

Even in the absence of neurodegenerative disease, the aging brain is subjected to global cortical atrophy and loss of functional integrity. Particularly affected structures are the frontal lobes (Kaup et al., 2011) and the hippocampus (Persson et al., 2006). Reduced cortical volume has also been demonstrated in the parietal cortex (Kalpouzos et al., 2012), and functional connectivity between parietal regions and prefrontal cortex has been shown to be reduced in aging (Madden et al., 2010). With these structures impacted in old age, it is not surprising that aging is associated with deficits in WM and attention in particular (Grady, 2012; Harada et al., 2013). Conversely, genetic markers of aging have been shown to accumulate more slowly in the cerebellum than in other parts of the brain (Liang and Carlson, 2020). Given the cerebellum’s role in precise motor timing (Bastian, 2006; Bares et al., 2007), this points to a possible preservation of timing abilities in old age. Additionally, the motor cortex does not belong to the heavily impacted structures in normal aging, but some atrophy in motor cortical regions has been observed, together with atrophy in the corpus callosum (Seidler et al., 2010). Functional neuroimaging has found different patterns of brain activation in motor tasks between older and younger people. More specifically, some motor tasks (especially fine motor control) engage motor regions in everyone, but additionally engage prefrontal and sensorimotor networks in aged people (Heuninckx et al., 2005, 2008; Seidler et al., 2010), even in cases where there are no age-related differences in performance. This might reflect a shift from more automatic to more controlled processing with age, in spite of the same task instructions and the same outcome (Heuninckx et al., 2005). In line with those results. a resting-state functional connectivity study also found a pattern of heightened connectivity in some motor networks (motor cortex and cerebellar lobule VIII with putamen) and decreased connectivity in others (cerebellar lobule V and VIII with sensorimotor portion of insular cortex; Seidler et al., 2015). All these results might point to a mechanism of compensation, in which motor cortex pathology in old age is offset by an additional use of other domain-general neural resources, among others in the prefrontal cortex. This might also explain relations between sensorimotor function and cognitive functions in age (Li and Lindenberger, 2002). If motor tasks rely more on prefrontal cortex and other networks in old age, we would assume a covariation of motor performance and cognitive functions, and competition of resources if a task involves both motor and cognitive requirements. Alternatively, the additional involvement of non-motor regions in older people might also reflect a less efficient use of neural resources in older people (Stevens et al., 2008; Grady, 2012).

This picture of age-related changes in cognitive functions and neural mechanisms generates some predictions. If we imagine internal clock in terms of a pacemaker emitting regular pulses and a person’s SMT as “one tap every *n* pulses,” and if we suppose that internal clock slows with age, as has often been suggested (Vanneste et al., 2001; Turgeon and Wing, 2012), this means that the rate of regularly emitted pulses is lower with age. Consequently, SMT and preferred perceptual tempo should be lower with age, but we do not have a reason to believe that people would tap spontaneously with a higher variability. If one chooses their own tempo and provides one tap every *n* pulses, even if these pulses occur less frequently, variability is not expected to be affected. On a cerebral level, the relative preservation of cerebellar integrity (Liang and Carlson, 2020) also suggests preserved variability in spontaneous tapping, given the cerebellum’s role in predictive movement control (Bastian, 2006). However, on fast unpaced tapping tasks, we expect a slower speed as well as reduced consistency. As mentioned above, CV when tapping as fast as possible has been linked to attention (Colella et al., 2021), and we expect lower available attentional resources in aging to be reflected in lower consistency.

Regarding paced tapping, due to the relative preservation of cerebellum and motor cortical structures in aging, we do not expect a great decline in performance in elderly people, at least at intervals that are neither very fast nor very slow, except perhaps for very old people. With regard to synchronization to very fast stimuli, we might imagine lower consistency. If an internal pacemaker emits fewer pulses with age, this should lead to a reduced temporal resolution of perceived stimuli and therefore increasing difficulty to synchronize to them as interval length decreases. Conversely, due to the asserted use of the cognitively controlled timing system with very slow intervals (above 1 s), we would expect differences in brain activation with aging. As the memory and attentional resources might already be used for fundamental motor synchronization due to compensatory rewiring, we hypothesize a larger involvement of parietal areas and prefrontal cortex to fulfill those requirements, and/or a decrease in performance.

### Neurocognitive Disorders

In order to describe the neuropathology of NCDs and make predictions regarding SMS in cognitively impaired people accordingly, it is important to acknowledge that NCD is a complex clinical picture that can have several different etiologies, including AD, vascular NCD, frontotemporal NCD, NCD with Lewy bodies, and others.

Most of the brain structural damage in NCDs, especially in AD, occurs in the hippocampus and surrounding parietal–temporal areas, even in early stages of the disease (Braak and Braak, 1991; Scheff et al., 2006; Jacobsen et al., 2015; Rémy et al., 2015). Besides the hippocampus, there is reduced structural and functional integrity in the prefrontal cortex (Braak and Braak, 1991; Rémy et al., 2015). WM and attention capacity are reduced, beyond what is usual with healthy aging. Conversely, primary sensory, motor, visual and anterior cingulate cortices are relatively well preserved (Jacobsen et al., 2015). However, despite cortical atrophy affecting some structures more than others, the entire cortex is affected and particularly in late stages of the disease, motor areas show the same neurofibrillary tangles and neurotic plaques as other areas, as some autopsy studies reveal (Golaz et al., 1992; Suva et al., 1999). Some research even suggests that motor cortex atrophy occurs in early stage NCD, although motor symptoms are visible only in later stages of the disease. Similarly to healthy aging, there is some evidence for compensatory processes: One study reported hyperexcitability of the sensorimotor cortex in AD patients compared to age-matched controls, even in the absence of motor symptoms (Ferreri et al., 2016). A diffusion tensor imaging study demonstrated some rewiring with alternative connectivity between motor cortex and other cortical and subcortical areas in AD and MCI (Agosta et al., 2010). Additionally, this level of rewiring was correlated with hippocampal atrophy and AD-related changes in grey matter volume. It is conceivable that these NCD-related changes reflect an attempt to compensate for degeneration of motor structures by employing additional brain networks to perform motor tasks.

Even AD can be considered a somewhat heterogeneous disease that could possibly be further divided into subtypes (Lam et al., 2013). In vascular NCD, the second most common type, the brain damage depends on the location of the vascular accident. It can be primarily cortical, primarily subcortical, or a combination, and the neuropsychological profile is accordingly variable (Braaten et al., 2006; O’Brien and Thomas, 2015). In short, NCD is a diverse clinical picture, and even its subtypes can further be divided into subcategories, so it is difficult to make predictions with regard to SMS performance. The following predictions, as well as the results discussed later, might to some extent be generalizable, but apply to AD patients more than to people with other NCDs.

Generally, there is not too much evidence to suggest that people with NCDs would perform worse on unpaced tapping tasks. However, given that self-paced tapping has been shown to depend more on dorsolateral prefrontal cortex and parietal lobe integrity (Witt et al., 2008), it could be that accuracy and consistency might be lower in people with NCDs. Additionally, it must be mentioned that unpaced tapping might be differently affected by different patterns of neural degeneration. For example, basal ganglia pathology is particularly associated with vascular NCD (Hansen et al., 2015; Banerjee et al., 2017), so maybe people with vascular NCDs, and particularly those with damage to the basal ganglia, might be more impacted in unpaced tapping tasks, which may rely more heavily on basal ganglia (Schwartze et al., 2011), as discussed above.

Regarding paced tapping tasks, as with healthy aging, the degree to which people with NCDs might be impaired might especially depend on interval length. Since motor structures, including the cerebellum, are relatively well preserved in people with NCDs, we expect relatively good performance when using the automatic timing network, that is, at fast and comfortable intervals, and especially at tasks using synchronization without continuation, that is, without a requirement to create a mental representation of a given interval. We might expect some difficulty, reflected in higher variability in people with NCDs compared to healthy participants on synchronization–continuation tasks, since continuation tapping involves WM and structures like the prefrontal cortex, parietal and temporal lobes are implicated, all of which are more impacted in NCDs than in healthy aging. Besides higher variability, we hypothesize people with NCDs to speed up on continuation tapping at slow tempi, given that speeding up on such tasks has previously been related to performance on WM tasks (Henley et al., 2014). We could conjecture that tapping at slow tempi would be even more impaired in people with frontotemporal NCD, since here the prefrontal cortex is especially impaired. Perhaps strategies like explicit counting and subdivision that can help people to synchronize with slow sequences are also less utilized by people with NCDs than by their healthy counterparts.

## Sensorimotor Synchronization in Healthy Aging

In the following, we discuss and synthesize some of the relevant research that features tasks that make people of different ages tap in a rhythmic fashion, to test the predictions we made. We report separately on unpaced and paced tapping tasks, which both contribute complementary information about how temporal mechanisms change with age. Previous research has demonstrated that people (regardless of age or cognitive impairment) spontaneously produce intervals of around 600 ms (Dalla Bella et al., 2017b) and that synchronization to external rhythms is best between 400 and 800 ms (McAuley, 2010), thus suggesting a relationship between these two measures in the sense that synchronization consistency and accuracy may become smaller as the difference between target tempo and internal tempo becomes greater.

### Unpaced Tapping

#### Spontaneous Motor Tempo

Studies that have compared SMT across age groups generally agree with each other in that they find a significantly slower tempo in older compared to younger people (Vanneste et al., 2001; Baudouin et al., 2004; McAuley et al., 2006; Turgeon and Wing, 2012; see Table 1). It is merely the magnitude of slowing with age, as well as the exact developmental course, that were somewhat different across studies. For example, while some studies found quite substantial differences in the ITIs of participants of different ages (1,072 ms for old participants; 654 ms for young participants; Baudouin et al., 2004), this difference is smaller in other studies (747 ms in old participants; 536 ms in young participants; Vanneste et al., 2001) and even subtler in others (McAuley et al., 2006, found the SMT of people aged 75+ to be 648 ms, 632 ms for participants between 60 and 74, and 522 ms for people aged 39–59).

**Table 1:** Studies investigating spontaneous motor tempo in different age groups.

Study		Young	Middle-aged	Old	Very old
Baudouin et al. (2004)	*n*	20	–	21	21
	*M*_Age_ (SD)	25.05 (3.71)		73.19 (4.54)	85.90 (3.32)
	SMT in ms (SD)	654***^a^ (186)		1,072***^a^ (318)	1,125***^a^ (426)
McAuley et al. (2006)	*n*	119	52	25	21
	Age range	18–38	39–59	60–74	75–95
	SMT in ms (SD)	630**^b^ (22)	522**^b^ (34)	632**^b^ (59)	648**^b^ (43)
**Age significantly predicted SMT** **	
Turgeon and Wing (2012)	*n*	60			
	*M*_Age_ (SD_Age_)	54.35 (25.18)			
	SMT				
**Age significantly predicted SMT** ***		
Vanneste et al. (2001)	*n*	8	–	11	–
	*M*_Age_ (SD)	26.25 (1.83)		69 (4.52)	
	SMT in ms (Range)	536** (283–727)		747** (625–1,035)	

M, mean; SD, standard deviation; SMT, spontaneous motor tempo.

a Old and very old groups significantly different than young group; no difference between old and very old.

b No statistical test for between-group differences was performed, but a regression analysis found age to significantly predict SMT.

** *p* < 0.01;

*** *p* < 0.001.

Regarding the developmental course, some authors demonstrated that it is especially in very old age that a slowing of SMT is visible. McAuley et al. (2006) found a cubic relationship between age and SMT, suggesting that this variable slows particularly late in life (i.e., after the age of 75). Similarly, in study of Turgeon and Wing (2012), a slowing of spontaneous motor rate was visible particularly in participants aged 75 and above.

Consistency of spontaneous tapping, which is usually expressed in terms of CV, does not appear to be as affected by age, as we predicted. McAuley et al. (2006) and Vanneste et al. (2001) found that old and very old people at their preferred tempo tapped as consistently as young ones. Along similar lines, using linear regression, Turgeon and Wing (2012) did not find age to predict a significant proportion of variance of CV. Thus, at a tempo that participants choose themselves, differences in terms of consistency have not been observed between young and old people.

As SMT reflects one’s natural rate of rhythmic motor activity, it is often thought of as being related to preferred perceptual tempo. McAuley et al. (2006) investigated the relationship between SMT and preferred perceptual tempo, in a study in which they presented rhythmic sequences of different speeds to their participants and asked them to judge whether each sequence was too fast, too slow, just right (relative to their favorite speed), or anything in between. They found that preferred perceptual tempo slowed with age along with SMT and that these two variables were highly correlated. One may then conclude that SMT, which appears to significantly slow down with age, might reflect the slowing of one’s internal clock in old age.

#### Fastest and Slowest Unpaced Tapping

Other types of unpaced tapping are those that require participants to tap in a repeating, continuous fashion as fast or as slow as possible, to see at which upper and lower limit participants are able to maintain a regular tap. As demonstrated by a large number of studies, when given the instruction to tap regularly as fast as possible, older people tapped at a slower rate than younger people (Nagasaki et al., 1988; Cousins et al., 1998; McAuley et al., 2006; Turgeon et al., 2011). This fastest tempo may slow down most evidently from the age of about 70 years onwards (McAuley et al., 2006). Conversely, McAuley et al. (2006) also made their participants tap as slowly as possible at a constant rate, in which case older people sped up more than younger people. It appears, then, that aged people have a narrower range of tempi at which they can consistently produce taps than young people.

The variability of taps at a fastest regular speed has not always been investigated. Where it has, CV was shown to be affected by aging, although this effect was weaker than the effect of aging on interval size. More specifically, Turgeon et al. (2011) found age to account for 7% of the variance in CV scores and for 32% of the variance in ITI, which might reflect decreased attention in old age.

### Paced Tapping

#### Synchronization Without Continuation

In tasks requiring participants to synchronize their tapping with an external regular signal, differences between young and old have been less clear than in the unpaced tapping tasks described above (see Table 2). First of all, several studies did not find differences between these two groups in terms of variabilities or accuracies (Krampe et al., 2005; Drewing et al., 2006; Turgeon et al., 2011). Contrary to that, Nagasaki et al. (1988) did find CV to correlate with age at all the intervals examined (between 200 and 1,000 ms), but did not find a significant effect of age on asynchronies.

**Table 2:** Studies investigating paced tapping in different age groups.

Study		Fast tempo (<350 ms)		Comfortable tempo		Slow tempo (>1,000 ms)	
		IOI	Age effect	IOI	Age effect	IOI	Age effect
Bangert and Balota (2012)	Consistency	–		500 ms/1,000 ms	No diff.	1,500 ms	O < Y***
	Accuracy				No diff.		O < Y*
Carment et al. (2018)	Consistency	333 ms	No diff.	500 ms/1,000 ms	No diff	–	
	Accuracy		O < Y***		No diff.		
Drewing et al. (2006)	Consistency	333 ms	No diff.	999 ms	No diff.	–	
	Accuracy		No diff.		No diff.		
Duchek et al. (1994)	Consistency	–		550 ms	No dif.	–	
	Accuracy			550 ms	No diff.		
Krampe et al. (2005)	Consistency	300 ms	No diff.	400 ms/600 ms/800 ms/1,000 ms	No diff.	1,200 ms/1,600 ms/2,000 ms	No diff.
	Accuracy		No diff.		No diff.		No diff.
Krampe et al. (2010): Single-task condition	Consistency	–		550 ms	No diff.	2,100 ms	No diff.
	Accuracy				No diff.		No diff.
Dual-task condition	Consistency				O < Y***		O < Y*
	Accuracy				O < Y*		O < Y**
McAuley et al. (2006)	Consistency	150 ms/225 ms/337 ms	No diff.	506 ms/759 ms	No diff.	1,139 ms/1,709 ms	No diff.
	Accuracy		No diff.		No diff.		O > Y
Nagasaki et al. (1988)	Consistency	200 ms/250 ms/333 ms	O < Y*	500 ms/1,000 ms	O < Y*	–	
	Accuracy		No diff.		No diff.		
Thompson et al. (2015)	Consistency	–		500 ms/667 ms	O < Y***	–	
	Accuracy				O < Y*		
Turgeon et al. (2011)	Consistency	–		600 ms/900 ms	No diff.	–	
	Accuracy				No diff.		
Vanneste et al. (2001)	Consistency	300 ms	No diff.	400 ms/500 ms/600 ms/700 ms	No diff.	–	
	Accuracy		No diff.		No diff.		

For simplification, paradigms using synchronization with continuation and synchronization–continuation are reported together here. O, old participants; Y, young participants; IOI, inter-onset interval.

* *p* < 0.05;

** *p* < 0.01;

*** *p* < 0.001.

One study (Thompson et al., 2015) did report differences between old and young participants in both variabilities and accuracies. In this study, older adults (age range 51–80) tapped to a regular beat with a higher variability and with a larger asynchrony than younger and middle-aged adults (age range 18–43). These results may seem surprising, especially in light of the relatively young age of their old adults group (*M*_Age_ = 63.67; for comparison, Drewing et al., 2006 did not find such age-related effects in the group of people aged 78–88). However, the difference found by Thompson et al. (2015) may, at least in part, be explained by musical experience. Since the authors were interested in how musical experience influenced beat synchronization, they recruited many musically experienced participants (in the young and middle-aged adult groups, there were 32 people with and 11 people without musical background). However, musical background was not assessed in the group of older adults, and therefore old and young adults may have not been matched on this variable. In fact, the respective asynchronies found in this study (around 40 ms before the beat for the older group and around 15 ms before the beat for the younger groups) are remarkably close to values that have previously been found in research comparing musicians and non-musicians (Aschersleben, 2002). Assessing musical training in all groups, including older adults, would have made it possible to verify whether young and middle-aged adults’ superior performance was due to musical experience rather than age, and also to investigate whether musical training could be a neuroprotective factor in aged people.

Up until now, all studies we discussed here described paradigms in which people synchronized their movements to an isochronous sequence of repeating single beats. And in fact, while a large part of those studies discussed their results in terms of their relevance for music perception and cognition, one could argue that their stimuli were not strictly musical. People move spontaneously to a musical beat (Leman et al., 2017), even though this beat is a perceptual construct that does not have a clear physical correlate. Beats sometimes co-occur with musical notes, but not necessarily: A beat can occur on a silent event (McAuley, 2010). Extracting a beat from a complex auditory signal, such as music, while automatic, might be a quite different process than merely synchronizing to an explicit beat. And therefore, this process might be differently affected by aging and NCDs. Indeed, rhythm difficulties might be more easily identified with music than with simple, repeating tones (Sowiński and Dalla Bella, 2013; Falk et al., 2015). Another reason to look at music in addition to metronomic sequences is that generally, people tend to tap with a higher asynchrony (that is, their taps precede the corresponding stimulus onset) when synchronizing with a metronome compared to music (Thaut et al., 1997; Aschersleben, 2002), a result that has recently been confirmed in elderly people (Ghilain et al., 2020a,b), but still lacks a definitive explanation. It has been suggested that subdivision of intervals between beats leads to a reduction of negative asynchronies and their variability, and that synchronization may be facilitated by the recurrence of different pitches, event duration, or intensities (Repp, 2003). In this way, music could be seen as an extreme case of subdivision, and the observed pattern of reduced asynchronies in tapping to music might be no different from what has been observed in tapping to a subdivided metronomic sequence. In either case, however, no studies exist to the best of our knowledge that compare old and young participants on an SMS task requiring participants to tap along with an auditory complex, that is, musical, stimulus.

#### Synchronization–Continuation

In synchronization–continuation tasks, participants first synchronize with a metronome and then continue tapping at the same rate when the sound has stopped. Generally, only the performance in the continuation phase is analyzed (Wing and Kristofferson, 1973). Regarding the question of how older adults perform in this paradigm, the results are somewhat mixed (see Table 2). Turgeon et al. (2011) used a synchronization–continuation paradigm in which the target tempi per individual were determined based on one’s SMT in the preceding spontaneous tapping task (see Section “Spontaneous Motor Tempo”). At the intervals IOI = 600 ms and IOI = 900 ms, age did not significantly predict variability or accuracy.

Other studies also did not find any differences in ITI or variability on synchronization–continuation tasks using tempi between 300 and 700 ms (Duchek et al., 1994; Vanneste et al., 2001). One study found intact performance in old subjects even at intervals as short as 150 and 225 ms (McAuley et al., 2006), but a higher asynchrony (speeding up) at the slowest target interval (1,709 ms) in their oldest group (75 years and above), but not in the group of 60–74-year-olds. Conversely, Carment et al. (2018) reported an increase in variability among older subjects at the IOI of 333 ms, but not at 500 and 1,000 ms. Another study found differences in both variability and accuracy among older participants at a target interval of 1,500 ms, but not at 500 ms or 1,000 ms (Bangert and Balota, 2012). These results lend support to the purported existence of two systems involved in temporal perception, an automatic and a cognitively controlled one, the latter of which is particularly implicated in the processing of slow intervals and presumably used less efficiently by older people.

Krampe et al. (2010) conducted a study with a dual-task design in which participants were required to tap to a faster (IOI = 550 ms) or slower (IOI = 2,100) tempo, while performing variants of the NBack WM task (Dobbs and Rule, 1989). In this task, participants were exposed to a sequence of visual stimuli and were asked to indicate when the current stimulus matched the one presented two steps earlier in the sequence. While this paradigm falls a bit outside the order of simple synchronization–continuation paradigms presented here, it is relevant for two reasons. First, the authors also report performance under single-task conditions, that is, while performing synchronization–continuation without a concurrent second task. In this case, there was no difference in variability or accuracy among age groups, even at the slow tempo of IOI = 2,100 ms, which is in contrast to the speeding up among oldest subjects in study of McAuley et al. (2006) study and the lower consistency and accuracy observed in oldest participants of Bangert and Balota (2012). This discrepancy might in part be explained by age differences: While older participants of Krampe et al. (2010) had a *M*_Age_ of 67, healthy old participants of Bangert and Balota (2012) were on average 75 years old and McAuley et al. (2006) included only people aged 75 or older in their oldest group. Therefore, it indeed appears to be difficult at least for very old people to synchronize successfully with a fading stimulus presented at a slow tempo (of at least an IOI = 1,200 or more).

The second reason for which study of Krampe et al. (2010) study is relevant to this review is that the dual-task nature of the paradigm might provide relevant information regarding the cognitive mechanisms involved in tapping at slow frequencies. Dual-tasking caused people regardless of age to speed up at the slow tempo, but additionally caused old participants to speed up at the fast tempo. Similarly, variability was significantly higher in older adults (at fast and slow tempi), but only in the dual-task condition. The authors’ interpretation is that maintaining temporal precision and stability, even at a tempo of IOI = 550 ms, might be a quite automatic process in younger people, but might cost older people more attention and WM resources, in line with the compensation hypotheses mentioned above. Without any cognitive load, they can deploy that attention and WM resources to perform the task as well as their young counterparts. However, with fewer of these resources available, their performance will drop. Indeed, as we discussed in Section “Brain and Cognitive Correlates of Healthy Aging and Neurocognitive Disorders,” it may be the cumulative effect of slow tempo, cognitive load, and age-related competition for prefrontal resources due to compensatory rewiring of the motor system that is expressed in the lower performance of older people.

#### Conclusion: Sensorimotor Synchronization in Healthy Aging

Perhaps the clearest finding regarding aging and SMS is a lower SMT in older people, which has been demonstrated in people in their 60 s and above but may be most apparent from the age of around 75 years onward. Similarly, preferred perceptual tempo appears to slow with age and to correlate with SMT. In contrast, variability appears to be intact in old participants tapping at a self-chosen speed. Moreover, the range of rates at which aged participants can tap regularly is narrower than for young participants, with a slower fastest tempo and a faster slowest tempo.

Paced tapping tasks have not tended to reveal differences in variability or accuracy between old and young subjects. Exceptions are synchronization–continuation tasks where participants had to maintain a relatively slow rate beyond 1 s, in which older people tended to speed up and tap less consistently, especially very old people from around 75 years old. Higher variability has also been reported at fast intervals of 333 ms and below and some research points to an interaction between age and cognitive load even at intervals that are close to people’s natural pace.

## Sensorimotor Synchronization in Neurocognitive Disorders

### Unpaced Tapping

#### Spontaneous Motor Tempo

Having discussed the relative slowing of SMT with age, we now discuss how this variable is affected by NCD. A few sources have suggested that unpaced tapping may become more variable and SMT may slow in NCDs, especially in advanced stages (see Table 3). Roalf et al. (2018) found people with AD and MCI to tap with a higher variability compared to age-matched healthy controls, and variability was negatively associated with their score on the Mini-Mental State Examination (MMSE; Folstein et al., 1983), a widely used test for screening cognitive function among the elderly. People with AD produced significantly fewer taps during 1 min of tapping at a comfortable rate compared to healthy participants, whereas the speed of participants with MCI did not significantly differ from either of the other groups. The difference in produced ITI between the AD group and healthy older adults was significant, albeit not very large (469 and 441 ms, respectively). Similarly, Rabinowitz and Lavner (2014) found that in a group composed of patients with MCI and patients with a diagnosis of dementia, variability was higher in that group than in healthy controls. Moreover, patients tapped at a slower speed (747 ms) than their healthy counterparts (581 ms). Additionally, MMSE score was found to be correlated with mean ITI, suggesting a slowing of SMT with disease progression. In contrast, some other studies compared people with and without NCD on a spontaneous motor tapping task and did not find any differences in ITI or variability (Martin et al., 2017; Ghilain et al., 2020a). In these studies, subjects tapped at a comfortable speed for 30 s or for 30 ITIs, respectively. The apparent disagreement in the literature may be explained by different instructions that were given. The durations in which people performed the SMT task were shorter in Roalf et al. (2018; six blocks of 10 s) and Rabinowitz and Lavner (2014; one block of 15 s). In just 10 or 15 s, even when told to tap at a comfortable speed, people might feel pressure to produce as many taps as possible. Indeed, the produced ITIs were relatively low, even compared to the ITIs found in the studies on SMT in healthy aging (see Table 1). Although speculative, it may be that people with NCD react to this pressure differently than healthy older adults. Additionally, cognitively impaired people might potentially have had more problems understanding the task instructions. If, due to lack of clarity of the task requirements, people with NCD tapped more hesitantly (slower and more variably) in the beginning of a task, this would be reflected in their overall scores more heavily if the whole trial was just 10 or 15 s long.

**Table 3:** Studies investigating spontaneous motor tempo and spontaneous tapping in people with and without neurocognitive disorders.

Study		Healthy	Mild NCD	Major NCD
Ghilain et al. (2020a)	SMT in ms (SD)	Between-group difference in SMT: n.s.		
	Consistency	Between-group difference in CV: n.s.		
Martin et al. (2017)	SMT in ms (SD)	820.33 (237.68)	–	935.88 (381.72)
	Consistency	*Not computed*		
Rabinowitz and Lavner (2014)	SMT in ms (SD)	581***	747***	
	Consistency	*Not computed*		
Roalf et al. (2018)	*n*	131	46	62
	SMT in ms (SD)	438*^a^ (67)	468*^a^ (102)	468*^a^ (91)
	Consistency (IIV)	0.72*^a^	0.83*^a^	0.82*^a^

NCD, neurocognitive disorder; SMT, spontaneous motor tempo; SD, standard deviation; IIV, intra-individual variability; CV, coefficient of variation; n.s., not significant.

a Major and mild NCD groups significantly different than healthy group; no difference between major and mild NCD.

* *p* < 0.05;

*** *p* < 0.001.

Therefore, the SMT tasks used in Ghilain et al. (2020a) and Martin et al. (2017), affording their participants more time to establish a regular tapping pattern reflective of their internal speed, might be a better representation of their real SMT. Indeed, the rates observed in their studies, which lay roughly between 700 and 950 ms, are close to SMT values that have been observed in physiological aging (see Table 1). It therefore does not appear that during spontaneous tapping, ITI and consistency deteriorate in NCD, at least not in the mild to moderate stages of NCD that participants in all the studies quoted above tended to be in. More research, particularly including people with more severe NCD, is needed to establish an effect or absence of effect of NCD on SMT.

#### Fastest Unpaced Tapping

As with healthy aging, some studies have looked into the variability and speed at which people with major and mild NCD tap when asked to tap regularly as fast as possible.

Under these conditions, Kluger et al. (1997) identified no difference in terms of produced tempo between patients with MCI and age-matched healthy participants, but found people with mild AD to tap at a lower speed. In contrast, Goldman et al. (1999) did not find an effect of AD on produced fastest tempo. Variability was not taken into account in these studies. Colella et al. (2021) did find tapping variability to increase in people with MCI, but they did not find a difference in tempo between the groups. Taken together, these studies suggest that while a decrease in fastest tempo at which people can tap is only seen in advanced stages of NCD, regularity of fast tapping already appears to decrease in people with mild NCD. This is an interesting parallel to the results found by Roalf et al. (2018), who also observed decreased variability in people with MCI but decreased absolute tempo only with a diagnosis of AD in their SMT task.

To the best of our knowledge, there do not exist any studies comparing people with and without NCD on the ability to tap in a regular fashion as slowly as possible. Since paced tapping at a slow tempo might rely more heavily on WM and attention, we might infer that people with NCD, who tend to be impaired in these domains, might speed up and/or tap with a higher variability when instructed to keep a regular pace as slowly as possible, a prediction that remains to be tested.

### Paced Tapping

#### Synchronization Without Continuation

The performance of tapping along with an auditory metronome has been investigated in people with NCDs in few studies. There might be several reasons for this, but we presume that many of the tasks described in the last few sections could not have been conducted in the same fashion with people with NCDs, especially in groups of patients with major NCD. In this group, particular attention must be paid to avoid stressful, unpleasant, artificial, and invasive laboratory situations. The listening and movement production tasks described in the previous sections might not be suitable for this patient group, and instead research with multimodal stimuli that creates a social or quasi-social situation might be conducive here (Desmet et al., 2017; Lesaffre et al., 2017). An example of this is the paradigm we described in Ghilain et al. (2020a,b), and Hobeika et al. (2021) in which participants were instructed to tap along with music or a regular metronome (IOI = 800 ms) while a musician, either seated across from the participant or projected onto a life-sized screen, vocalized and tapped along with the same stimulus. Under these conditions, benefitting from the presence of a musician, no differences in asynchrony or variability were found between people with and without NCD (see Table 4). It must be mentioned that the patients in this study were recruited from a day hospital rather than an inpatient care facility, so they might reflect a relatively independent and mildly impaired NCD group. Their average MMSE score of 20 was just on the fringe between mild and moderate cognitive impairment (Folstein et al., 1983), so we cannot exclude the possibility that a sample of more heavily cognitively impaired patients would show deficits in tapping performance compared to healthy older adults. The lack of effect of NCD might also be explained by other methodological variables, such as the use of only one tempo (close to elderly people’s SMT, see Table 1) or the impact of social entrainment related to the presence of a partner during the task. Since this paradigm is adapted to people with major NCD, it would be interesting to have a group of more cognitively impaired people perform this task.

**Table 4:** Studies investigating paced tapping in people with and without neurocognitive disorders.

Study		Fast tempo (<350 ms)		Comfortable tempo		Slow tempo (>1,000 ms)	
		IOI	NCD effect	IOI	NCD effect	IOI	NCD effect
Bangert and Balota (2012)	Consistency	–		500 ms/1,000 ms	NCD < healthy^**^^b^	1,500 ms	NCD < healthy^*^
	Accuracy				No diff.		NCD < healthy^**^
Carment et al. (2018)	Consistency	333 ms	NCD < healthy^***^	500 ms/1,000 ms	No diff.	–	
	Accuracy		No diff.		No diff.		
Duchek et al. (1994)	Consistency	–		550 ms	No diff.	–	
	Accuracy				No diff.		
Ghilain et al. (2020a)	Consistency	–		800 ms	No diff.	–	
	Accuracy				No diff.		
Henley et al. (2014) synchronization without continuation	Consistency	–				1,500 ms	NCD < healthy^*^^a^
	Accuracy						No diff.
Synchronization–continuation	Consistency						NCD < healthy^*^
	Accuracy						NCD < healthy^*^^a^
Martin et al. (2017)	Consistency	–		*Determined by SMT task*	NCD < healthy^*^	–	
	Accuracy				No diff.		
Nichelli et al. (1993)	Consistency	–		1,000 ms	NCD < healthy^***^	–	
	Accuracy				Healthy < NCD^*^^c^		

For simplification, paradigms using synchronization without continuation and synchronization–continuation are reported together here. IOI, inter-onset interval; NCD, neurocognitive disorders; SMT, spontaneous motor tempo.

a Behavioral variant frontotemporal dementia, but not Alzheimer’s disease (AD).

b At 1,000 ms, but not at 500 ms.

c AD patients were slower than elderly controls, but since elderly controls tended to underestimate the target interval, AD patients’ responses were actually more accurate.

* *p* < 0.05;

** *p* < 0.01;

*** *p* < 0.001.

Besides this, there do not exist many studies evaluating SMS with complex auditory (musical) stimuli, although, as we argued in Section “Synchronization Without Continuation,” it is not only interesting to directly compare SMS to metronomic sequences and SMS to music, due to the presumably different mechanisms involved in beat extraction, but providing musical stimuli can also be of particular value in creating an experimental situation that will make people with NCDs feel comfortable and that has some ecological validity.

One study that did uncover NCD-related differences in SMS was that by Henley et al. (2014). In this study, participants with different variants of frontotemporal dementia and AD synchronized with a metronome at an IOI of 1,500 ms. This study did not find differences in accuracy but higher variability in participants with behavioral variant frontotemporal dementia, but not in participants with other variants of frontotemporal dementia or AD, compared to healthy age-matched adults.

#### Synchronization–Continuation

Bangert and Balota (2012), whose study was mentioned in Section “Synchronization–Continuation” about healthy aging, had their participants tap at a rate of 500, 1,000, and 1,500 ms, after the metronome stopped. Besides comparing the performance of young and healthy old participants, they also compared the latter group’s performance to that of people with very mild (mean MMSE = 27) and mild dementia (mean MMSE = 24). At the interval of 500 ms, they identified no group differences in terms of variability or accuracy (see Table 4). At 1,000 ms, people with dementia tapped with a greater variability but at the same accuracy as their healthy counterparts, whereas at 1,500 ms both consistency and accuracy were reduced in cognitively impaired people. These effects were stronger in people with mild compared to very mild dementia. Complementing these findings, Carment et al. (2018) found unimpaired performance with MCI and AD (mean MMSE = 22) at tapping rates of 500 ms and 1,000 ms, but increased variability at the most rapid tempo of 333 ms. Duchek et al. (1994) employed an IOI of 550 ms and found merely a non-significant trend toward higher variability. Nichelli et al. (1993) did not test their participants at shorter intervals, but found AD patients’ variability to be larger than healthy participants’ at an interval of 1,000 ms, corroborating findings of Bangert and Balota (2012). Along similar lines, Henley et al. (2014), in a synchronization–continuation task at 1,500 ms, found lower accuracy (speeding up) and greater variability in people with behavioral variant frontotemporal dementia, and greater variability in people with AD compared to healthy participants. Based on these studies, it appears that ITI and/or variability in people with NCD are only impacted at relatively long or relatively short intervals, presumably due to an impacted cognitively controlled timing system. However, it is important to point out that the participants in all these studies had on average very mild or mild NCDs.

In contrast, the patients in the study by Martin et al. (2017) tapped with a higher variability than healthy controls, even at a comfortable rate (in this study, the synchronization–continuation task was performed at a rate that depended on their SMT in the preceding spontaneous tapping task; see Section “Spontaneous Motor Tempo”). The apparent discrepancy between this study and the ones mentioned before might be explained by degree of impairment: The patients in study of Martin et al. (2017) had an average MMSE of 19, which indicates moderate cognitive impairment and which was lower than the MMSE scores reported in any of the studies mentioned above. Taken together, these results suggest that SMS performance in people with mild NCD might decline only at relatively long or short tempi, whereas in more severe cognitive impairment, people might tap less precisely even at tempi that are close to their natural speed. Nonetheless, more research about timing abilities in people with moderate or even severe cognitive impairment is needed.

#### Conclusion: Sensorimotor Synchronization in Neurocognitive Disorders

Overall, there is not a lot of evidence suggesting that tapping at a comfortable rate reveals differences between people with and without NCD. On the other hand, tasks requiring regular tapping as fast as possible have demonstrated increased variability even for people with mild NCD and increased variability as well as slower tapping in people with major NCD. The fact that changes in variability during unpaced tapping are visible even in people with mild NCD are relevant in terms of using motor tasks as a diagnostic tool: While motor speed might only reliably identify people with major NCD, tapping variability could be used to identify people who are at a risk for developing a more serious impairment. Indeed, in MCI, motor impairments are predictive of developing AD (Aggarwal et al., 2006; Ghilain et al., 2020a).

Presently, there is little research studying the effect of cognitive impairment on performance in simple tapping (synchronization without continuation) tasks, but the extant literature suggests that people with frontotemporal NCD might tap less precisely than AD patients and healthy controls at a tempo of 1,500 ms. More research is needed with more different tempi and different levels of cognitive impairment. More commonly, synchronization–continuation studies have been used, and suggested that people with NCD might speed up and show increased variability at slow IOIs from around 1,000 ms, as well as show increased variability at short IOIs below 500 ms. There is some suggestive evidence that even at a comfortable rate, people with a moderate or severe NCD might tap less precisely. Etiology of the NCD may play a role, with a possible interaction between tempo and type of NCD, but more research is needed with various groups of patients with NCD due to different causes, and different levels of impairment.

In short, there is a need for more research investigating SMS abilities in people with moderate and severe cases of NCD. At the same time, however, designing tasks adapted to people with NCD is not trivial: a great deal of effort has to be done to ensure people understand the instructions and to create experimental paradigms that are not stressful, invasive, or unpleasant to the participants. With the existing research, differences between people with and without NCD must be evaluated critically: It is important to verify that they tap into the mechanisms a study claims to test, or whether observed differences may reflect differences in motivation, comfort, or having well understood the instructions. Finally, a fruitful avenue of research would be more studies using musical stimuli of different levels of rhythmic complexity, not only to test the ability to infer and maintain an implicit beat, but also to create a situation that is engaging and motivating, ideally involving a social or quasi-social element resembling real-life musical interaction (Desmet et al., 2017; Lesaffre et al., 2017; Ghilain et al., 2020a,b; Hobeika et al., 2021).

## Future Directions

To move forward, we suggest the following methodological considerations when conducting research about SMS abilities in healthy aging and NCDs. First, it is useful to employ designs that compare healthy young, healthy old, and cognitively impaired old participants in the same study, in order to disentangle effects of age and of neurodegenerative disorder. A few studies discussed here have done that (Bangert and Balota, 2012; Carment et al., 2018), but most have not. Second, the literature reviewed here suggests that age effects on SMS abilities may not be linear, so it is particularly important to include sufficient amounts of participants from the latest decades of life. Third, likewise, with regard to NCDs, etiology (AD, vascular NCD, frontotemporal NCD, etc.) and severity seem to play important roles when investigating their effects on SMS. We are aware of only one study that compared people with different types of NCDs (Henley et al., 2014). In terms of severity, it is valuable to compare people with major and mild NCDs, or to define neurocognitive impairment as a continuous variable, measured by rating scales, such as the MMSE, mentioned above, the Montreal Cognitive Assessment (Nasreddine et al., 2005), or the Mattis Dementia Rating Scale (Mattis, 1976). These scales need not be mutually exclusive but can be complementary instruments of evaluation of cognitive state (Freidl et al., 1996; Cullen et al., 2007; Arevalo-Rodriguez et al., 2013). Fourth, when conducting research with people with NCDs, particular attention should be paid to creating a pleasant and stimulating atmosphere and to avoid stress. Otherwise, it cannot be excluded that observed between-group differences are reflective of differences in motivation, discomfort, or comprehension of the instructions, rather than sensorimotor abilities. Finally, musical training should always be probed, to ensure that different participant groups are matched on this variable but also to test the possible rehabilitative or neuroprotective effect of music engagement in old age and NCD.

As we mentioned, it will be interesting to conduct more studies on SMS to music. SMS is about predicting, extracting, and maintaining a representation of a beat; however, this beat is not a physical entity but a cognitive percept that needs to be extracted, a process that might be quite different for real music than for isochronous metronomic stimuli. The question of whether aged people and/or people with NCDs have a particular difficulty or a particular ease synchronizing with a musical rhythm is at present still an open one.

Furthermore, this review discussed tasks in which participants produced rhythmic responses, but there also exists task assessing beat perception in a purely perceptual fashion, such as the Harvard Beat Alignment Test (Fujii and Schlaug, 2013) and some subtests of the BAASTA (Dalla Bella et al., 2017b). Moreover, rhythm production and perception can independently be impacted (Bégel et al., 2017). It would be interesting to test rhythm perception abilities specifically in elderly people with and without NCDs.

Since the focus of this review was SMS to simple isochronous or musical rhythms, we did not touch upon the topic of error correction. Although error correction is an ever-present process in SMS without which one would eventually become out of sync (Vorberg and Wing, 1996), it is usually examined by introducing perturbations of the IOI and testing how quickly and efficiently participants adapt their own tempo to them. Generally, a difference is made between phase correction and period correction, The former refers to an automatic, often unconscious mechanism of adaptation to subtle perturbations that is associated with primary and secondary somatosensory cortical activity, whereas the latter refers to a mechanism correcting for more obvious changes in the temporal sequence that depends on attention and awareness and involves brain networks, such as the basal ganglia, prefrontal, medial frontal, and parietal regions (Thaut et al., 1998; Repp, 2001; Praamstra et al., 2003; Repp and Su, 2013; Ross et al., 2018). It is conceivable that phase and period correction would be differently influenced by aging and NCDs. A study (Repp and Keller, 2004) that manipulated attention by introducing a concurrent mentally taxing task found period correction to be affected by this reduction in available attentional resources, whereas period correction was not. Furthermore, in a recent study with musicians (Versaci and Laje, 2021), attention was guided in a more explicit way by directing some participants’ attention to temporal features of the task, in turn yielding higher accuracy and more efficient resynchronization after a perturbation of a relatively large size, probably employing period correction mechanisms. It can be conjectured that very old and old people would be especially impaired in tapping along with sequences containing tempo changes, particularly those large enough to depend on period correction mechanisms, and, in cerebral terms, on frontal and parietal integrity.

## General Conclusion

Neurocognitive disorders, as well as healthy aging, are often associated with a decline in memory, attention, and executive function. Less often, we tend to think of sensorimotor dysfunction as a prototypical symptom of physiological and pathological aging, except in the case of Parkinson’s disease. Restricting our review to the other etiologies of NCDs, we have indeed observed many examples in this review in which no clear difference on SMS performance emerged between young, healthy old, and/or cognitively impaired old people. However, SMS abilities are a complex set of skills with different subcomponents that may be differently affected by aging and NCDs, and substantial heterogeneity exists between and within different NCDs, as well as between aging brains in general.

In some cases, there is clear evidence for decline on SMS tasks for elderly people and/or people with NCDs. Specifically, SMS ability might be especially impaired above the age of around 75 and for people with moderate and/or severe NCDs. People with frontotemporal NCDs might particularly struggle with SMS tasks, although research comparing different NCD etiologies is sparse. These effects might be augmented with stimuli whose tempo is further away from a person’s comfortable, natural tempo (i.e., IOIs faster than around 300 ms and slower than around 1,000 ms). The exact instructions, protocol, and trial length might play a role, especially in spontaneous motor tasks. Musical background might also modulate the effect of aging and NCDs on SMS abilities, and cognitive load might have a different effect on performance for the different groups. More specifically, due to rewiring and compensation mechanisms, SMS, usually a quite spontaneous process requiring little cognitive resources, might become heavier in cognitive load and interfere with other concurrent tasks. Rhythmic complexity might be relevant although our analysis was restricted to two ends of the extreme: simple, isochronous rhythms, and music, the latter of which merits more attention than it is currently given. To fully understand late-life development of sensorimotor processes, more research is needed that reflects the wide array of types of auditory stimuli as well as the diversity of aging brains.

The observation that slightly younger elderly people and people with mild NCDs tend to be relatively unimpaired in synchronizing particularly at tempi of around 500 and 1,000 ms is a strong argument for the use of music-based interventions. Musical tempi tend to lie within this range, and capitalizing on this relatively preserved skill could be a fruitful approach. The stimulation of motor systems in older adults across different levels of cognitive impairment may not only improve their sense of rhythm, but lead to emotional and cognitive benefits as well as improvements in the ability to predict the unfolding of events in the environment, leading to improved communication and reduced social isolation.

## Author Contributions

AS, LH, and SS: conceptualization. AS: writing the original draft. LH and SS: review, editing, and supervision. DH-G: review. All authors contributed to the article and approved the submitted version.

## Funding

Funding has been received through the European Union’s Horizon 2020 research and innovation program under the Marie Skłodowska-Curie grant agreement no 847568. Moreover, this publication was supported by the French government through the Programme Investissement d’Avenir (I-SITE ULNE/ANR-16-IDEX-0004 ULNE) managed by the Agence Nationale de la Recherche.

## Conflict of Interest

The authors declare that the research was conducted in the absence of any commercial or financial relationships that could be construed as a potential conflict of interest.

## Publisher’s Note

All claims expressed in this article are solely those of the authors and do not necessarily represent those of their affiliated organizations, or those of the publisher, the editors and the reviewers. Any product that may be evaluated in this article, or claim that may be made by its manufacturer, is not guaranteed or endorsed by the publisher.

## Footnotes

^1^ https://www.nitrc.org/projects/mricrogl/
